# In Regard to Otelea et al.

**DOI:** 10.4274/balkanmedj.galenos.2019.2019.5.140

**Published:** 2019-08-22

**Authors:** Muhammet Gürdoğan, Servet Altay

**Affiliations:** 1 Department of Cardiology, Trakya University School of Medicine, Edirne, Turkey

To the Editor,

Otelea et al. (1) reported in one of your recent issues their study findings on the cross-sectional correlation between the leptin-to-adiponectin ratio and unfavorable plasma lipid profile in 93 predominantly nonobese, healthy young adult women. The researchers reported a significant relationship between high leptin–adiponectin ratio and cardiometabolic risk indicators such as anthropometric measurements, plasma atherogenic index, lipid accumulation product, and insulin resistance in the healthy young adult population, while no relationship has been reported between low-grade inflammatory markers such as interleukin-6 and high-sensitivity C-reactive protein. These findings suggest that high leptin–adiponectin ratio may be a good indicator in the evaluation of cardiovascular and metabolic risk in the nonobese healthy young population (1).

In recent studies, it has been reported that there is a strong relationship between leptin and adiponectin levels and insulin resistance and metabolic syndrome, and adipokines can be used as a diagnostic marker for metabolic syndrome (2,3,4). In addition, it has been suggested that leptin–adiponectin ratio is a better marker of insulin resistance and metabolic syndrome than leptin or adiponectin only (2,3). In this study by Otelea et al. (1), there are no data on how the leptin–adiponectin ratio changes with gender and the effect of this change on cardiometabolic risk indicators. However, in the literature, leptin and adiponectin levels and hence the leptin–adiponectin ratio were reported to differ between genders (3,4,5). In a study by Chou et al. (4), leptin and adiponectin levels and the leptin–adiponectin ratio were significantly higher in women than men. The increase in leptin levels in women is attributed to the higher proportion of fat tissue in women compared with men, whereas the decrease in adiponectin levels in men is explained by the androgens inhibiting adiponectin secretion (3,4,5).

In light of this information, considering the differences between gender may help to better clarify this issue in future studies that investigate the relationship between the adipokine profile and the cardiometabolic risks.
